# 

**Published:** 1913-10-04

**Authors:** 


### Appointments.

Mr. W. Lupton, B.A.Cantab., M.R.C.S., L.R.C.P., of St. Thomas's Hospital, has been appointed second house surgeon at Addenbrooke's Hospital, Cambridge, and took up his duties on Monday, the 22nd ult.

Mr. H. F. Rutherford, who has just completed three years' apprenticeship in the secretary-superintendent's office at Addenbrooke's Hospital, Cambridge, has now been appointed on the permanent staff as in-patient and stores clerk.

Mr. George Heaton, M.A., M.B., B.Ch. (Oxon), F.R.C.S. (England), has been appointed honorary consulting general surgeon to the Birmingham and Midland Eye Hospital. Three assistant honorary surgeons were also appointed: Mr. T. Harrison Butler, M.A., M.D., B.Ch. (Oxon); Mr. R. Beatson Hird, M.D., Ch.B. (Birm.), F.R.C.S. (Edin.); and Mr. D. Priestley Smith, M.B., Ch.B. (Birm.), M.R.C.S. (Eng.), L.R.C.P. (Lond.). Mr. Harrison Butler is the honorary ophthalmic surgeon to the Coventry and Warwickshire Hospital, and has acted as oculist to the Coventry Education Committee. His past appointments also include that of assistant surgeon at the British Ophthalmic Hospital, Jerusalem. Mr. Beatson Hird is honorary ophthalmic surgeon to both the General Hospital and Maternity Hospital, Birmingham. He was recently appointed clinical lecturer in ophthalmic surgery to the University of Birmingham. Mr. D. Priestley Smith, whose father was the first professor in ophthalmology at Birmingham University, has had experience at the eye department of the Queen's Hospital and at the Government Eye Hospital at Madras.

### Gifts and Bequests.

Mr. John Price, of Wimbledon, has left £1,000 to the London Hospital and £500 each to the Westminster Hospital, the Wimbledon Hospital, and Shrewsbury Infirmary.

Lady Hardinge has received the sum of 10,63,000 rupees (over £70,000) towards the proposed Women's Medical College and Hospital at Delhi says, the Reuter correspondent at Simla. The Ruling Princes of Jaipur, Haidarabad, Kotah, Baroda, the Maharani of Hutwa, and the Jodhpur Durbar are announced as the principal subscribers.

Mr. Walter Pewtress Appleton, of Forest Hill, S.E., has bequeathed £40,000 upon trust for his wife for life, and on her death he left, from this trust fund, £500 each to Dr. Barnardo's Homes, Spurgeon's Orphanages, Mr. Groom's Homes for Crippled Children, the Forest Hill Day Nursery, the Home for Sick Children, Lower Sydenham, and the Boys' and Girls' Homes, Perry Vale and Dartmouth Road, Forest Hill, S.E.

Miss Hannah Elizabeth Gartside, of Holmfirth, Yorks, has left £1,000 each to King Edward's Hospital Fund for London, Brompton Hospital for Consumption, St. Thomas's Hospital, and Guy's Hospital; £500 each to Dr. Barnardo's Homes and the Association for the General Welfare of the Blind; £1,900 to other charitable institutions; and the residue of her estate, subject to specific bequests, in equal shares to Guy's Hospital and St. Thomas's Hospital.

Miss Harriet Anne Sobieska Sneyd, of Rhyl, has bequeathed £1,000 upon trust for the benefit of one of her servants for life, with remainder to the Royal Alexandra Hospital, Rhyl.

The Swansea Hospital Building Extensions Fund during August has received the following:—D. M. Glasbrook, £1,000; John Glasbrook, £1,000; R. J. Matthews, £105; Burton Livingstone, £105; Richard Jenkins, £25; Anonymous, £10 10s.

Amongst the latest contributions received for King Edward's Hospital Fund for London are the following annual subscriptions:—Messrs. Lloyds Bank Limited, £250; Messrs. Cox and Co., £100; the London Joint Stock Bank (Limited), £100; Colonel Robert Gordon-Gilmour, £26 5s.; Sir Edward Stern, £25; Mr. J. G. A. Baird, £25; Mrs. E. Crossley, £25; and Mr. Henry L. Hopkinson, £25.

### GENEROUS GIFT BY A FORMER CHAIRMAN.

Sir Edward Wood and the Board of Governors of the Leicester Royal Infirmary have received contributions of £500 from Mr. T. Fielding Johnson, and £100 from Mrs. T. Fielding Johnson, to the Children's Hospital Reconstruction Fund. Mr. Fielding Johnson is the oldest governor of the institution, and, in addition to serving on the board for thirty-one years, was chairman during the years 1898-1902. He is one of the vice-presidents of the institution and one of the trustees, and has been a generous benefactor to the institution. Mrs. Fielding Johnson, like her husband, has been for many years a member of the board of governors of the institution.

### THE DAISY DAY FUND AT MANCHESTER.

A pleasant function took place at Ancoats Hospital, Manchester, last week, when Councillor Grime, on behalf of the Daisy Day Fund Committee, presented the representatives of the Hospital Committee with a cheque valued £720, being the net proceeds of a Daisy Day and Carnival held in July last. In making the presentation, Councillor Grime expressed the wish of his committee that a Daisy Day bed be inaugurated. Mr. A. E. Gaddum, honorary secretary to the hospital, expressed his committee's willingness to fall in with their wishes in the naming of a bed.

